# “Y” Configuration of the Arterial Pedicle or the Use of a Saphenous Vein Graft for Microsurgical Reconstruction in the Old and Diseased—A Retrospective Study

**DOI:** 10.3390/jcm14010157

**Published:** 2024-12-30

**Authors:** Maximilian Moshammer, Andrzej Hecker, Nikolaus Watzinger, Anna-Lisa Pignet, Ron Martin, Gerlinde Weigel, Lars-Peter Kamolz, Werner Girsch

**Affiliations:** 1 Division of Plastic, Aesthetic and Reconstructive Surgery, Department of Surgery, Medical University of Graz, 8036 Graz, Austria; maximilian.moshammer@medunigraz.at (M.M.); werner.girsch@kabsi.at (W.G.); 2 COREMED—Centre for Regenerative Medicine and Precisions Medicine, Neue Stiftingtalstrasse 2, 8010 Graz, Austria; 3 Department of Plastic and Hand Surgery, Burn Unit, Trauma Center Bergmannstrost, Merseburger Strasse 165, 06112 Halle, Germany; 4 Austrian Armed Forces, Medical Center East, Medical Facility Vienna, 1210 Vienna, Austria

**Keywords:** free tissue transfer, microsurgical reconstruction, peripheral artery disease, vascular calcification, arterial patch, interposition graft

## Abstract

**Background:** Non-healing soft tissue defects pose challenges to treating physicians. Microsurgical reconstruction is a treatment option for achieving wound closure and limb salvage. These free tissue transfers are often challenging due to associated risk factors. This study aimed to evaluate microsurgical reconstruction using specialized microsurgical techniques for non-healing spontaneous or post-traumatic soft tissue defects in an elderly, high-risk patient cohort with peripheral artery disease. **Methods**: A retrospective study was conducted on patients with radiologically confirmed peripheral artery disease who underwent free tissue transfers between 2004 and 2010. Patients were included in whom one of two surgical techniques was used, including a “Y” configuration of the arterial pedicle, employed either as an interposition graft or as an arterial patch, or the use of a saphenous vein graft. Patient demographics, comorbidities, flap/limb survival, and surgical techniques were analyzed. **Results**: Twenty patients at a mean age of 68 (+/−9.3) years underwent 21 primary flap surgeries. Trauma-derived soft tissue defects were predominant (55%). Latissimus dorsi muscle flaps were most frequently utilized (52.4%). The flap success rate was 90.5% at a 12-month follow-up, with no secondary amputations recorded. The lost flaps were replaced by additional free tissue transfers without further complications. **Conclusions**: This study demonstrates the feasibility of free tissue transfers in high-risk patients with complex soft tissue defects and vascular calcifications. Thorough preoperative planning and the application of specialized surgical techniques are crucial for favorable outcomes in challenging clinical scenarios.

## 1. Introduction

The aging population in industrialized countries presents new challenges for their respective surgeons. Risk factors such as smoking, diabetes, hypertension, or hypercholesterolemia contribute to a higher prevalence of conditions such as peripheral artery disease (PAD), which now affects up to 5.6% of people worldwide [1]. In PAD, vessel calcification causes stenoses that restrict blood flow, leading to ischemic pain, non-healing ulcerations, and an increased risk of amputation [2,3]. Up to 80% of all leg ulcerations also involve a venous component caused by chronic venous insufficiency, resulting in difficult-to-heal wounds [4]. In vascular-compromised extremities, these non-healing soft tissue defects (STDs) can form spontaneously, after trauma, or due to exposure to pressure [5]. The prevalence of ulcers of various types, based on clinically detected ulcerations, is estimated to be between 1.51 and 2.21 per 1000 people and is on the rise [6,7]. Conservative treatment involves control of hyperglycemia, optimalization of the blood supply and nutrition, appropriate wound management, and offloading [8]. Free tissue transfer (FTT) provides an additional surgical option for closing defects in these patients [9,10]. The issue is that most of the risk factors contributing to PAD are also linked to a higher failure rate of FTT. Additionally, PAD is a significant factor contributing to the increased risk of amputation following FTT [11,12]. An especially challenging patient population consists of those with PAD and chronic limb-threatening ischemia (CLTI), who require revascularization prior to undergoing FTT [13]. The impact of CLTI on mortality and patients’ lives is comparable to that of cancer [14]. Meta-analyses outline the challenges that arise when performing microsurgery in a challenging patient collective. Flap survival following FTT to the lower extremities is described with 94% [15] but decreases to 88–90% when only diabetic foot ulcers are included [16,17]. Problems that arise in microsurgical reconstructions in calcified vessels could present as hard and inflexible vessels, small anastomotic leaks that are difficult to address, turbulent flow, and calcified sections in recipient or donor vessels, rendering them unsuitable for anastomosis altogether [18,19]. However, given that age is not a limiting factor for free flap procedures, the indications for FTT are broadening, and more information regarding the connection between PAD and FTT is needed [20,21]. The aim of this study was to evaluate the microsurgical treatment of non-healing spontaneous or posttraumatic STD in a high-risk patient cohort, specifically elderly patients with comorbidities and calcified vessels [22]. In a previous work, it was shown that limb salvage through femorodistal bypass and the use of the bypass vein graft for flap anastomosis is highly effective and produces stable limb conditions [23]. Here, results are presented concerning microsurgical defect coverage in patients who do not require bypass surgery at a distal level and where the original vessels are always used for anastomoses. Particular emphasis is placed on the surgical techniques employed to examine whether reconstructive procedures can yield satisfactory outcomes even in patients with severely compromised vascular status.

## 2. Materials and Methods

### 2.1. Patients

High-risk patients who underwent surgery via microsurgical FTT between January 2004 and December 2010 were retrospectively reviewed. Surgical techniques included a “Y” configuration of the arterial pedicle, used either as an interposition graft or as an arterial patch, or a saphenous vein graft. Patients with radiographically confirmed PAD were defined as high-risk patients. All patients were operated on by the same plastic surgeon. The study was approved by the Institutional Review Board of the City of Vienna, Austria (EK-14-043-VK, 18 March 2014). Data regarding following areas were retrieved from patient records: age at the time of surgery, gender, affected limb, cause of the respective defect, preoperative radiographs, relevant comorbidities, preoperative vascular reconstruction, flap-type, surgical technique, and flap/limb survival at a 12-month follow-up. Computed tomography angiography, magnetic resonance angiography, or digital subtraction angiography of the upper extremity or the leg and pelvic region was conducted in all patients as part of the preoperative diagnostic assessment. Each radiograph revealed severe vasculopathy with at least one crural artery remaining patent down to the ankle. The indication for free tissue transfer in each patient was a defect with exposed functional structures requiring coverage with well-vascularized tissue. The free flap transfers were always performed as separate interventions. Beginning with the debridement of the wound, the optimization of arterial blood flow followed if necessary. Then, a VAC (Vacuum-Assisted Closure) therapy was installed. The debridement of the wound was either performed separately and earlier or simultaneously with the optimization of arterial blood flow. If patients underwent bypass surgery, a femoropopliteal bypass or iliofemoral bypass was performed, with the intervention always conducted at proximal levels above the knee. The recipient vessel for the free flap was always the original vessel distal to the bypass.

### 2.2. Surgical Technique

**Figure 1:**
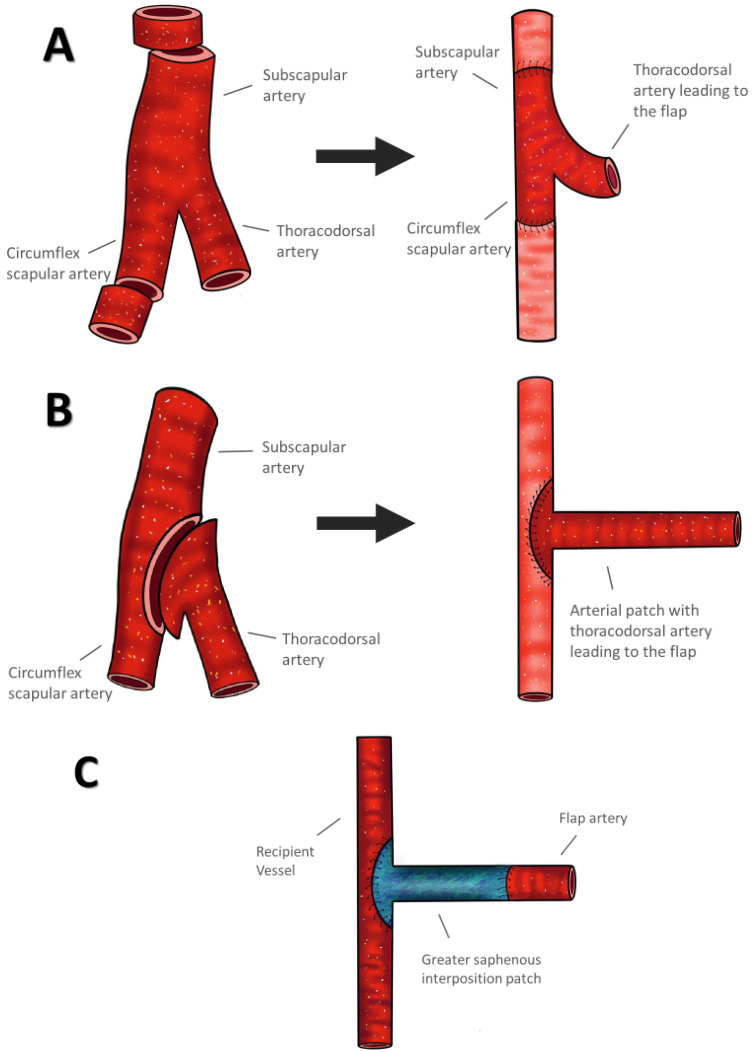
Microsurgical techniques. (**A**) Interposition of the “Y” anastomosis into the recipient vessel. (**B**) Insertion of the arterial patch with parts of the subscapular and the circumflex scapular artery into the recipient vessel. (**C**) Inserted venous patch with subsequently anastomosed flap artery.

All patients were operated on under general anesthesia, with vessel anastomoses performed using a microscope. The indication for the flaps used was determined based on both the size and depth of the defect. When employing a latissimus dorsi (LDM) or a serratus muscle flap (SM), the thoracodorsal artery was harvested along with segments of the subscapular artery and the circumflex scapular artery, resulting in a proximally configured “Y” pedicle. This configuration offered two options for anastomosis: An interposition graft or a patch. The pedicle with the “Y” configuration could serve as an interposition graft, inserted into the corresponding artery of the leg using either continuous or interrupted 7.0 sutures (Figure 1A). Sutures for the microvascular anastomosis were always non-absorbable. Alternatively, segments of the subscapular artery and the circumflex artery could be used as a patch for an end-to-side anastomosis, utilizing either continuous or interrupted 7.0 or 8.0 sutures in an inside-out manner, as shown in Figure 1B. When harvesting a bipedicled LDM flap, a perforator branch of the posterior intercostal artery was identified at the level of the sixth intercostal space and then anastomosed end-to-side. This allowed the flap to be designed as a myocutaneous flap with an inferior cutaneous extension, which was transferred with the LDM flap. In cases where a gracilis muscle flap (GM) or an anterior thigh flap (ALT) were utilized, a venous patch from the saphenous vein with a side branch was harvested before the interposition of the vein the valves were disabled with a valvulotome (surgical instrument designed for valvulotomy). The venous patch was then sewn into the recipient vessel using either continuous or interrupted 7.0 or 8.0 sutures in an inside-out manner (Figure 1C). The side branch of the venous patch was anastomosed end-to-end with the donor vessel using interrupted 8.0 or 9.0 sutures.

In instances where an arterial or venous patch was employed for an end-to-side anastomosis, suturing was consistently performed in an inside-out manner. This technique minimizes the risk of dislodging any calcified vessel segments through the tension of the suture directed from the inside to the outside of the vessel. Apart from the chosen flap, one or two flap veins were always anastomosed end-to-end to concomitant crural veins. After completing the anastomosis, the successful reperfusion of the flaps was intraoperatively monitored. To prevent bulkiness of the muscle flaps, tension was applied to restore the original fiber length while safeguarding the pedicle. Excessive muscle tissue was then removed, and the muscle flaps (GM, LDM, and SM) were covered with split-thickness skin grafts (STSGs). Here, the contralateral thigh served as donor site for STSGs. The donor areas were closed both deeply and superficially with absorbable sutures. After the operation was completed, the flaps were monitored by the clinical staff, including specially trained nurses, and any questions were addressed by the attending physician on duty. The anticoagulation regimen included 100 mg of Acetylsalicylic Acid and 4000 international units of Enoxaparin, both administered once per day.

### 2.3. Statistical Analyses

Statistical analyses were performed using Excel 2016 (Microsoft Corporation, Redmond, WA, USA). The analysis involved calculating the mean and standard deviation.

## 3. Results

### 3.1. Patient Characteristics

In total, 20 patients (40% male patients) with a mean age of 68 (+/−9.3 SD) years and 21 primary free flaps were included in this study. Based on the STD reasons, the patient cohort was further divided into patients with trauma-derived STDs and non-trauma derived STDs. Demographic data, relevant comorbidities, and the reason for reconstruction for the trauma-derived STDs are shown in Table 1 and for the non-trauma-derived spontaneous STDs in Table 2.

#### 3.1.1. Patients with Trauma-Derived Soft Tissue Defects

In the trauma-derived STD group, there were a total of 11 patients who received 11 primary free flaps and two secondary free flaps. Ten patients had defects in the lower extremity, and one patient had a defect in the upper extremity (large dorsal area of the right forearm). Among these 11 patients, five also suffered from insulin dependent diabetes mellitus (IDDM) in addition to PAD.

#### 3.1.2. Patients with Non-Trauma Derived Soft Tissue Defects

In the non-trauma derived spontaneous STD group, there were a total of nine patients with ulcerations in the lower limbs who received 10 primary free flaps. One patient had a cutaneous radiation syndrome, and another patient suffered from a vasculitis. Seven patients suffered from spontaneous ulceration due to their underlying PAD. Four of these seven patients received a femoropopliteal or rather an iliofemoral bypass, and one patient received bypass surgery on each lower extremity. The last two patients underwent percutaneous transluminal angioplasty of the external iliac artery.

### 3.2. Free Flaps

Among the included 21 primary free flaps, 18 were muscle flaps (85.7%), two were myocutaneous flaps (9.5%), and one was a fasciocutaneous flap (4.8%). Eleven (52.4%) comprised LDM, of which two were bipedicled LDMs with inferior cutaneous extensions. Seven (33.3%) were GM, two (9.5%) were SM, and one (4.8%) was an ALT. A total of 90.5% (*n* = 19) of the free flaps were successfully brought to primary healing without complications. Two flaps (9.5%), one GM and one ALT, had to be replaced due to total loss with additional free flap surgeries using free LDM, respectively, which then healed without any issues. Both flap losses occurred in the group with trauma-derived STDs. Successful healing of the flaps was documented, with no need for secondary amputation, no infection, and no ischemic events distal to the anastomosis caused by thrombosis.

## 4. Discussion

In this retrospective study, the feasibility of FTT in a high-risk patient cohort using specialized microsurgical techniques was investigated. This study included a diverse group of patients with both traumatic and non-traumatic spontaneous STDs, all of whom had significant vessel calcifications in preoperative angiograms. In this cohort, a primary flap-success rate of 90.5% was observed, with no secondary amputations on the operated limb. This study provides insights into the surgical techniques, the outcomes achieved, and the challenges encountered in these cases.

Hassan et al. [24] reported a mean age of 55 (+/−13.5 SD) years for free flap recipients in a multi-institutional study analyzing 37,177 patients, while the mean age in this study’s cohort was 68 (+/−9.3 SD) years. The prevalence of diabetes, a known risk factor for surgery-related complications such as wound healing disorders [25], was reported to be less than 11% in the multi-institutional study versus 45% in this study’s cohort. Furthermore, less than 1% had a history of revascularization due to peripheral artery disease versus 35% in this study’s cohort, highlighting the severity of the comorbidities in this study [24]. The success rate of FTT in patients with calcified vessels which underwent revascularization procedures before surgery varies in the literature between 87% and 95% [10,26,27,28]. Some authors have also described success rates of up to 100% in patients with STDs and only single vessel perfusion [29]. Conversely, other studies reported flap success rates of 76% in patients who would have required amputation if microvascular reconstruction had not been attempted [30]. However, in this study’s cohort of high-risk patients, a flap success rate of over 90% was observed. This is an especially satisfying success rate, as the two lost flaps could be replaced with secondary flaps, achieving a limb survival rate of 100% after free flap transfer, despite the higher risk of amputation following primary flap loss [31]. The variability of the previously stated success rates could be attributed to differing inclusion criteria, as PAD is a broad term, and the relatively small patient cohorts. To the authors’ knowledge, meta-analyses on severe PAD and microvascular reconstruction are lacking but necessary. In this study’s cohort, primary debridement was performed even when vascularization had not yet been optimized. The prioritization of removing necrotic tissue was carried out to immediately reduce the septic load and decrease the risk of secondary amputations while waiting for a revascularization procedure [32].

Of all the flaps utilized, the LDM flap was the most frequently employed, followed by the GM flap. Both are considered “safe” flaps with well-defined vascular supply and large caliber vessels, making them suitable for free tissue transfers in a challenging patient cohort [33,34]. Overall, 2 flaps out of 21 were lost, one ALT flap and one GM flap. Notably, both flap losses occurred in the trauma cohort and involved the use of a venous patch. This could be attributed to the type of trauma and the associated vascular damage. Flap transfers after traumatic injuries are accompanied by increased risks of complications [31]. The first patient, who lost the ALT, was admitted due to a chainsaw injury that affected not only soft tissue, but also crucial functional structures such as extensor tendons, and muscles. In combination with atherosclerosis of the upper extremity, this led to a complicated case [35]. The second patient, who lost the GM, suffered a bimalleolar fracture, which subsequently developed into a pseudarthrosis and osteomyelitis.

When using the LDM flap and the SM flap, the pedicle is harvested, including the subscapular artery, to create the “Y” configuration of the arterial pedicle. However, in 8 to 18% of cases, the subscapular artery as “Y” configuration is reported to be absent, with the thoracodorsal artery and the circumflex scapular artery arising directly from the axillary artery [36,37]. Kawamura et al. [38] also described this vascular anomaly in 3 out of 16 dissections (19%). In these situations, alternative branches from the thoracodorsal artery should be identified to achieve a “Y” configuration of the pedicle, or a venous interposition patch could be used. In this study’s cohort, good anastomotic patency and no vascular complications distal to the interposition graft were documented, which is consistent with results reported in the literature [38,39,40]. In two cases, the LDM flap was used in a bipedicled fashion. This is a viable option for larger defects, ensuring proper perfusion of the distal segments of the LDM while providing an additional cutaneous extension [41]. When using a GM flap or an ALT, a saphenous vein interposition graft with a side branch is utilized for end-to-side anastomosis. This technique has been described as a viable option for microsurgical anastomoses in calcified vessels [42,43]. The advantages include creating a non-calcified recipient vessel for the subsequent end-to-end anastomosis with the donor vessel, minimizing the risk of pedicle kinking if the donor vessel is rigid, and facilitating easier handling and orientation during anastomosis [43]. The indications for vein grafts often include insufficient pedicle length and extensive trauma to the recipient area [44]. Subgroup analysis with interposition vein grafts in primary procedures without salvage operations shows success rates of up to 95% [45]. Despite these advantages, one should take into account that vein grafts may be associated with a higher risk of thrombosis. However, this topic is still controversial, with the literature being inconclusive [44]. It is important to consider the techniques used in the context of the time, as the operations were carried out between 2006 and 2010. During this period, perforator-based fasciocutaneous flaps were not as widely used as they are today, as most of the flaps utilized in this study were muscle flaps [46]. Still, muscle flaps remain an important tool, especially when dealing with larger defects [47].

Microsurgery poses considerable challenges as it inherently meets all the conditions of Virchow’s triad, largely due to the cutting and suturing of pedicles, the clamping of vessels during microsurgical anastomoses, and the hemostatic changes induced by the surgical procedure itself [48]. Sutures of the internal elastic lamina are inevitable, but to minimize the risk of thrombosis, additional iatrogenic injuries to the intima must be avoided. Arteries should be thoroughly inspected for defects before performing the anastomosis [49]. Additionally, atherosclerosis is a known risk factor that surgeons should consider when performing reconstructive microsurgery [50]. A preoperative vascular evaluation is essential to potentially improve peripheral perfusion before surgery. When selecting a flap, it is important to choose safe and reliable options to avoid unnecessarily prolonging the operation time. For microsurgical anastomoses, it is ideal to select a portion of the recipient vessel with little to no visible plaque formation. Additionally, the artery’s lumen should be thoroughly examined, as plaques can create a false lumen. Clamps should be positioned in non-calcified sections of the vessel. Whenever possible, suture bites should be directed from the inside to the outside to minimize the risk of dislodging plaques and to improve visualization of the intima. After completing microsurgical anastomoses, it is essential to ensure that the pedicle is in a secure position to avoid kinking or pressure on the vessel [18,51,52,53]. These technical prerequisites should be followed when employing the previously stated surgical techniques to ensure a favorable outcome.

## 5. Limitations

The limitations of this study include its retrospective design and the small sample size. The surgical technique also varied depending on the flap used. When GM or ALT flaps were employed, a venous patch was used for reconstruction. Conversely, when the LDM flap or SM was used, the Y-shaped pedicle was utilized for the anastomosis. Moreover, the etiology (traumatic, non-traumatic) and location of the defects were heterogenous, and different structures (bone, tendon) were affected differently (with or without infection). Furthermore, the absence of a control group with standardized surgical techniques makes it difficult to compare the described techniques with other surgical approaches. As previously stated, fasciocutaneous flaps were not as widely used during the observation period as they are today. Nowadays, a surgeon might choose a different flap for covering a specific defect on the leg compared to what would have been used in the past, which also hinders comparability. Finally, the follow-up period is adequate to monitor the initial healing outcomes but does not provide long-term data on the recurrence of ulcers or secondary amputations. For future prospective studies, particularly those investigating limb reconstruction in PAD patients, clinical as well as patient-centric aspects should be considered. Physical function plays a crucial role in determining quality of life (QoL) when comparing limb reconstruction to amputation [54]. Monitoring QoL can provide valuable insights into the recovery process and long-term outcomes, particularly in comparison to amputation. Additionally, if vascular reconstruction is combined with FTT, comprehensive preoperative and postoperative assessments (e.g., Doppler ultrasound, ankle–brachial index, transcutaneous oxygen pressure) of the vascular status should be performed. These objective measures can enhance the understanding of vascular dynamics and their impact on surgical and clinical outcomes in a high-risk patient cohort with complex STDs and preoperative evidence of PAD.

## 6. Conclusions

This retrospective study demonstrates the feasibility of FTT using specialized microsurgical techniques in a high-risk patient cohort with complex STDs and preoperative evidence of vascular calcifications. The success rate of 90.5% in this study suggests that specialized surgical techniques can be effective even in challenging cases. In elderly patients with multiple comorbidities, reconstructive options become more limited. With these findings, it is hoped to provide valuable guidance for physicians seeking optimal treatment strategies in similar clinical scenarios. Patients with severe atherosclerosis who are at risk of limb amputation can especially benefit from the use of these specialized surgical techniques. Careful preoperative planning is strongly suggested to achieve the best possible clinical outcomes.

## Figures and Tables

**Table 1:** Patient characteristics with trauma-derived soft tissue defects, flap type, and reason for reconstruction.

Age	Gender	Comorbidities	Flap Type	Reason for Reconstruction
54	m	IDDM, PAD	LDM	Motorcycle accident, syndesmosis ligament rupture, defect on the foot dorsum
56	f	IDDM, PAD	GM	Achilles tendon infection 6 months post-suturing
64	f	PAD	LDM-b	Run over by a bus, tibia fracture, soft tissue avulsion of the entire lower leg
65	f	IDDM, PAD	GM	Bimalleolar fracture, plating, infection, metal removal
65	f	IDDM, PAD	LDM-b	Fracture of the lateral malleolus, avulsion of the entire lower leg
68	m	PAD	LDM	Motorcycle accident, open knee dislocation, tear of popliteal artery, fasciotomy
70	m	PAD	ALT/LDM	Chainsaw accident, STD on the extensor side of the forearm
72	m	IDDM, PAD	LDM	Septic arthrodesis
74	f	PAD	GM/LDM	Bimalleolar fracture with exposure of both malleoli, osteomyelitis
79	f	PAD	GM	Necrosis of the skin and Achilles tendon, failure of initial defect coverage with distally pedicled sural and peroneal flap
86	m	PAD	LDM	Open tibia fracture

IDDM: insulin dependent diabetes mellitus; PAD: peripheral artery disease; Complex: complex defect with bone, tendon/muscles and soft tissue involvement; GM: gracilis muscle flap; LDM: latissimus dorsi; LDM-b: bipedicled latissimus dorsi flap.

**Table 2:** Patient characteristics with non-trauma derived soft tissue defects, flap type and reason for reconstruction.

Age	Gender	Comorbidities	Flap Type	Reason for Reconstruction
54	f	Radioderm, PAD	LDM	Schwannoma, resection and radiotheraphy, radiodermatitis, ulceration, osteomyelitis calcaneus
57	f	Vasculitis, PAD	GM	Vasculitis, ulceration of the distal lower leg
60	m	IDDM, PAD	Byp + LDM	Ulceration of the right heel
60	m	IDDM, PAD	Byp + LDM	Ulceration of the left forefoot
61	f	IDDM, PAD	PTA + SM	Ulceration of the Achilles tendon
66	f	PAD	Byp + GM	Ulceration of the lateral malleolus
69	m	PAD	Byp + LDM	Ulceration of the achilles tendon
78	f	PAD	Byp + GM	Ulceration of the distal lower leg
79	m	IDDM, PAD	Byp + LDM	Ulceration of the distal lower leg
79	f	IDDM, PAD	PTA + SM	Ulceration of the forefoot

IDDM: insulin dependent diabetes mellitus; PAD: peripheral artery disease; GM: gracilis muscle flap; LDM: latissimus dorsi; SM: serratus muscle flap; Byp: bypass at proximal level of the lower extremity; PTA: percutaneous transluminal angioplasty.

## Data Availability

The original contributions presented in the study are included in the article material; further inquiries can be directed to the corresponding author.
